# Responses to others’ pain in adults with autistic traits: The influence of gender and stimuli modality

**DOI:** 10.1371/journal.pone.0174109

**Published:** 2017-03-20

**Authors:** Jing Meng, Zuoshan Li, Lin Shen

**Affiliations:** 1 School of Education, Chongqing Normal University, Chongqing, China; 2 School of Mathematical Sciences, Chongqing Normal University, Chongqing, China; Leiden University, NETHERLANDS

## Abstract

Individuals with autism-spectrum disorder (ASD) exhibit impairments in response to others’ pain. Evidence suggests that features of autism are not restricted to individuals with ASD, and that autistic traits vary throughout the general population. To investigate the association between autistic traits and the responses to others’ pain in typically developing adults, we employed the Autism-Spectrum Quotient (AQ) to quantify autistic traits in a group of 1670 healthy adults and explored whether 60 participants (30 males and 30 females) with 10% highest AQ scores (High-AQ) would exhibit difficulties in the responses to others’ pain relative to 60 participants (30 males and 30 females) with 10% lowest AQ scores (Low-AQ). This study included a Visual Task and an Auditory Task to test behavioral differences between High-AQ and Low-AQ groups’ responses to others’ pain in both modalities. For the Visual Task, participants were instructed to respond to pictures depicting others’ pain. They were instructed to judge the stimuli type (painful or not), judge others’ pain intensity, and indicate the unpleasantness they personally felt. For the Auditory Task, experimental procedures were identical to the Visual Task except that painful voices were added. Results showed the High-AQ group was less accurate than the Low-AQ group in judging others’ pain. Moreover, relative to Low-AQ males, High-AQ males had significantly longer reaction times in judging others’ pain in the Auditory Task. However, High-AQ and Low-AQ females showed similar reaction times in both tasks. These findings demonstrated identification of others’ pain by healthy adults is related to the extent of autistic traits, gender, and modality.

## Introduction

Empathy is the ability to understand others’ emotional state in the context of the self [1–3]. It plays a crucial role in human social communication and interactions [2, 4]. Impairments of empathy have been suggested to be central to ASD [5].

In our everyday life, we are often faced with other people’s suffering. Perceiving others’ pain generally elicits empathic responses in typically developing (TD) individuals [6], however, previous studies found that individuals with ASD exhibited impairments in response to others’ pain. In an early study, researchers recorded how ASD participants reacted when an experimenter feigned an injury (e.g., banged her thumb). They found that while nineteen of the ASD participants attended to the experimenter’s injury, only two of them reacted with emotional responses [7]. Neural studies also indicated that when viewing pictures or video clips of others experiencing pain, ASD participants exhibited reduced unpleasantness [8] and impaired social understanding [9] relative to control groups.

Recent evidence suggested autistic traits measured by the AQ [10] were distributed across the population, and individuals with ASD scored at the extreme end of this distribution [11]. In support of this view, studies have found similar behavioral patterns in TD individuals with High-AQ and individuals with ASD [e.g., 12].

Previous studies have documented the association between autistic traits and the selective impairment in response to others’ feelings. For example, one study found that autistic-like social communication difficulties were associated with poorer recognition of emotions from social motion cues in both genders, but were associated with poorer facial emotion recognition in boys only [13]. However, no study has explored the responses to others’ pain of individuals with autistic traits.

Evidence has suggested that empathy in different domains is underpinned by a multimodal processing ability [14], and there are broad-ranging deficits in empathic processing in ASD individuals in both the auditory and visual modalities [15]. Understanding others’ pain in real life involves interpreting a variety of cues that include tone of voice, vocal content, gestures, and posture. Recognizing pain from human voices is the auditory equivalent of visual injury recognition. To this day, the majority of the empirical investigations of empathy for pain in individuals with ASD have focused on the visual modality. To our knowledge, no study has investigated the processing of others’ pain in TD individuals with autistic traits across a broad range of social signals.

In the present research, we explored the potential relationship between the responses to others’ pain and the autistic phenotype in TD adults. To provide a better understanding, we used both pictures and voices depicting others’ pain in our study. Based on the findings of previous research, we predicted that individuals’ levels of autistic traits would be associated with their responses to others’ pain, and participants with High-AQ would show poorer performance in response to others’ pain than controls.

## Methods

### Participants

Initially, a total of 1670 university students in Chongqing province, China, aged 17–24 years (Mean = 19.3 years, SD = 2.0 years) were recruited for our research. They completed a Mandarin Version [16] of the AQ questionnaire [10] to estimate the amount of autistic traits each participant revealed. From this sample, a subset of participants were identified as either “High-AQ” or “Low-AQ” according to their AQ scores (10% highest or 10% lowest scores correspondingly). Finally, 60 participants (30 males, 30 females) from the High-AQ group and 60 participants (30 males, 30 females) from the Low-AQ group were selected to take part in our research (see Table 1). This research was approved by the Chongqing Normal University research ethics committee. All participants had signed informed consent after being given a complete description of the study. The ethics committee approved this consent procedure.

**Table 1:** Age and AQ scores of High-AQ and Low-AQ groups.

Group	Gender	Age			AQ Score		
		Mean±SD	t (df = 58)	*p*	Mean±SD	t (df = 58)	*p*
**High-AQ**	**male**	19.00±1.14	-1.361	0.179	28.50±1.33	-0.428	0.671
	**female**	19.40±1.13			28.67±1.67		
**Low-AQ**	**male**	19.47±1.10	1.310	0.195	9.93±2.13	-0.914	0.364
	**female**	19.10±1.06			10.33±1.09		

Note. AQ = Autism Spectrum Quotient. *p*-values associated with an independent samples t-test on the differences of ages and AQ scores between males and females.

### Stimuli

#### Visual stimuli

**Fig 1:**
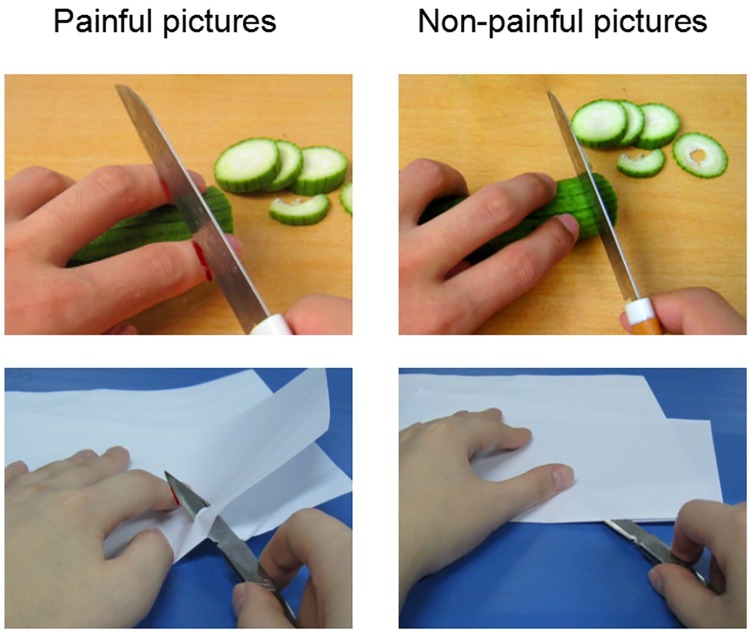
Examples of painful (left panel) and non-painful pictures (right panel).

The Visual Task consisted of 60 pictures (30 painful and 30 non-painful). These pictures were selected from our picture database previously validated and used in published studies [17, 18]. Each picture depicted a familiar event that can occur in everyday life. Each painful picture showed one model’s hands, forearms, or feet in a painful situation caused by accident, e.g., one hurt himself with a knife. Non-painful pictures corresponded to those painful pictures without any painful component. Luminance, contrast, and color were matched between painful and non-painful pictures, as illustrated in Fig 1.

#### Auditory stimuli

We selected 15 Chinese sensory painful words (e.g., hurting, cutting, aching; revised from Yang, et al., [19]) and 15 Chinese sensory non-painful words (e.g., wet, warm, cold) as filler material. The auditory stimuli used in our study were these Chinese words uttered by two speakers (one male and one female) in either painful or neutral emotional tones of voice respectively, with a presentation duration of 638–1000 ms (volume: 70–85 dB SPL). Thus, 60 voices (30 painful and 30 non-painful) were used in the Auditory Task. S1 Appendix provides voices used in the study with corresponding English translations. Before the study, 30 independent judges (undergraduate students) rated the voices for pain intensity (1 = no sensation, 4 = pain threshold, 9 = unbearable pain), emotional valence (1 = very happy, 9 = very unhappy), arousal (1 = extremely peaceful, 9 = extremely exciting). Descriptive statistics for the auditory stimuli are summarized in Table 2 for each dimension.

**Table 2:** Descriptive statistics of auditory stimuli (Mean±SD).

Dimension	Painful voices	Non-painful voices	t (df = 58)	*p*
**Pain intensity**	6.02±1.19	2.65±0.87	11.404	<0.001
**Valence**	6.64±0.71	5.74 ±1.24	3.442	0.001
**Arousal**	5.25±0.49	5.30±0.41	-0.420	0.676
**Duration (ms)**	817.17±87.55	809.07±81.50	0.371	0.712

Note. Mean and SD values for painful and non-painful voices, and *p*-values associated with an independent samples t-test on the differences between groups.

### Procedure

**Fig 2:**
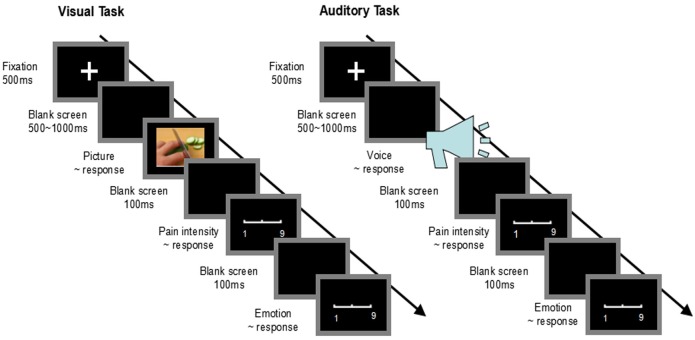
Flowchart describing experimental designs. Left column: Procedure of Visual Task. Right column: Procedure of Auditory Task.

A quiet room was used as the experimental place. Participants completed an informed consent form before engaging in the following experiment. A Visual Task and an Auditory Task were created and run on an IBM computer. Each task started with an instruction slide, followed by a training session. Participants were then instructed to do the main experiment. The order of the two tasks was counterbalanced. In both tasks, the test items were presented in random order. The procedures of the Visual Task and the Auditory Task are illustrated in Fig 2. Stimulus presentation was controlled using E-Prime (1.1).

#### Visual task

Prior to the task, an instruction slide was presented on the screen, participants were told that the computer screen would present a series of pictures, and they were instructed to respond with a key-press to each picture with their right hand to judge (1) the stimuli type (painful or not), (2) others’ pain intensity, (3) their own emotional reactions. Participants were instructed to complete the training session of 8 trials to become acquainted with the procedures. Four painful pictures and four non-painful pictures used in the training session were also selected from our picture database [17, 18] and were not used in the main experiment.

Each trial consisted of the following steps (see Fig 2):

1. A fixation cross was presented on a black screen with a duration of 500 ms.
2. 500–1000 ms later, a picture appeared, during which participants were instructed to respond as accurately and quickly as possible with a key-press (‘1’ or ‘2’) to judge whether the picture was painful or not (stimuli type judgment). The picture remained on the screen until a response was made. The key-press was counterbalanced across participants to control for order effects.
3. 1000 ms later, a 9-point pain intensity scale (1 = no sensation, 4 = pain threshold, 9 = most intense pain imaginable) appeared. Participants were required to provide a rating to judge others’ pain intensity based on the pain intensity scale via right-handed key-presses on a square keyboard pad with a 3×3 numeric display (pain intensity rating). The scale remained on the screen until a response was made.
4. 1000 ms later, a 9-point emotion scale (1 = extremely happy, 5 = neutral, 9 = extremely unhappy) appeared. Participants were required to provide a rating to judge their emotional reactions to the picture based on the emotion scale via right-handed key-presses on a square keyboard pad with a 3×3 numeric display (emotional reaction). The scale remained on the screen until a response was made.
5. An inter-trial interval of 1000 ms was presented before the start of the next trial.

The Visual Task was comprised of two blocks. For each block, 30 trials were randomly delivered. Each picture was presented only once in this task.

#### Auditory task

For the Auditory Task, experimental procedures were identical to the procedures of the Visual Task except that painful and non-painful auditory stimuli were used. Participants were instructed to do the training session of 8 trials (see S2 Appendix) to become acquainted with the procedure (Fig 2). Participants listened to the auditory stimuli through earphones. Participants were also required to respond with a key-press for stimulus type, pain intensity, and unpleasantness. The Auditory Task was comprised of two blocks. Painful and non-painful auditory stimuli were randomly presented, for a total of 60 trials. Each voice was presented only once in this task.

### Data analysis

Accuracies (ACCs) and reaction times (RTs) for stimuli type judgment, pain intensity ratings, and emotional reactions were calculated for each participant in each condition. Outliers with RTs outside Mean ± 3 SD were deleted (2.4% of all data). Statistical analysis was examined through repeated-measure analyses of variance (ANOVA) with two within-subject factors [Stimulus Type (Painful vs. Non-painful) and Modality (Visual vs. Auditory)] and two between-subject factor [Autistic Traits (High-AQ vs. Low-AQ) and Gender (Male vs. Female)]. Statistical differences were considered significant at a conventional *p* < 0.05 level. ANOVAs were followed by post hoc Tukey tests (*p* <.05).

## Results

ACCs, RTs, pain intensity ratings, and emotional reactions for both Visual and Auditory tasks in all conditions are summarized in S1 Table.

### ACC

**Fig 3:**
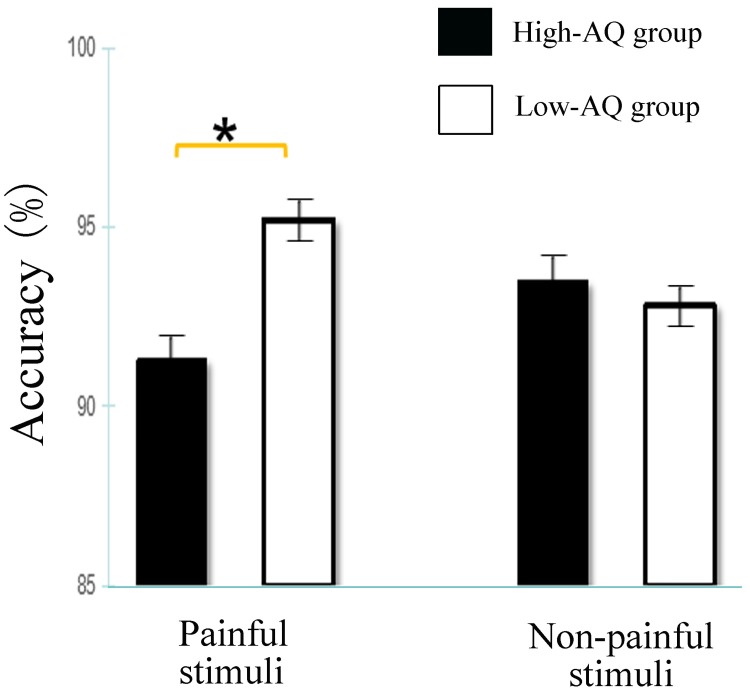
Bar graphs represent accuracy results of Stimulus Type × Autistic Traits interaction. Data are presented as mean values (more details see S1 Table). Error bars represent standard error of the mean. For painful stimuli, the Low-AQ group was more accurate than the High-AQ group. In contrast, for non-painful stimuli, the Low-AQ group was not different from the High-AQ group. Significant effect at p< 0.05 is marked by an asterisk.

In the ANOVA for ACCs, the main effect of Modality [F(1,116) = 14.531, *p* < 0.001, η^2^ = 0.111] indicated that participants were significantly more accurate in recognizing the visual stimuli (94.4±0.5%) than the auditory stimuli (92.0±0.6%). The Stimulus Type × Autistic Traits interaction [F(1,114) = 5.603, *p* = 0.020, η^2^ = 0.046], as illustrated in Fig 3, indicated that, for painful stimuli, the Low-AQ group (95.2±0.9%) was more accurate than the High-AQ group (91.3±1.1%) (p = 0.047). In contrast, for non-painful stimuli, the Low-AQ group (92.8±0.8%) was similar to the High-AQ group (93.5±1.1%) (p = 0.158). The interaction of Gender × Modality [F(1,114) = 8.465, p = 0.004, η^2^ = 0.068] indicated that females judged visual stimuli (95.2±0.7%) more accurately than auditory stimuli (91.0±0.9%) (*p* < 0.001), whereas ACCs for males were not different between visual stimuli (93.6±0.7%) and auditory stimuli (93.0±0.9%) (p = 0.521). No other main effect or interaction effect was significant (all p-values>0.05).

### RT

In the ANOVA for RTs, the main effect of Stimulus Type [F(1,116) = 30.251, p < 0.001, η^2^ = 0.207] indicated that RTs were significantly shorter for painful stimuli than for non-painful stimuli. The main effect of Modality [F(1,116) = 12.402, p = 0.001, η^2^ = 0.097] indicated that RTs were significantly longer for voices (1055.14±29.54) than pictures (940.44±32.80). Notably, the main effects were qualified by Autistic Traits × Gender × Stimulus Type interaction [F(1,116) = 8.979, p = 0.003, η^2^ = 0.072] and Autistic Traits × Gender × Stimulus Type × Modality interaction [F(1,116) = 5.855, p = 0.017, η^2^ = 0.048].

The simple effects analyses of Autistic Traits × Gender × Stimulus Type interaction indicated that High-AQ males judged painful stimuli (1009.24±47.95) slower than Low-AQ males (873.46±47.18) (p = 0.046), whereas no significant difference was found between (1) High-AQ males (1027.84±61.45) and Low-AQ males (1022.85±62.46) to non-painful stimuli (p = 0.955); (2) High-AQ females (952.96±49.64) and Low-AQ females (975.03±47.18) to painful stimuli (p = 0.748); (3) High-AQ females (1021.50±64.66) and Low-AQ females (1099.44±61.45) to non-painful stimuli (p = 0.384).

**Fig 4:**
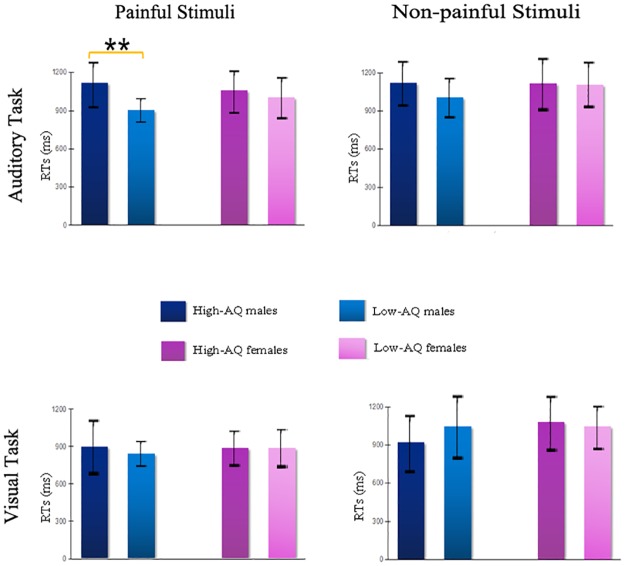
(Top) RTs for painful stimuli (left) and non-painful stimuli (right) in auditory tasks; (Bottom) RTs for painful stimuli (left) and non-painful stimuli (right) in visual tasks. Data are presented as mean values (more details see S1 Table). Error bars represent standard error of the mean. Significant effect at p< 0.01 is marked by two asterisks.

The Autistic Traits × Gender × Stimulus Type × Modality interaction, as illustrated in Fig 4, the simple effects analyses indicated High-AQ males judged painful voices slower than Low-AQ males in the Auditory Task (p = 0.007), whereas no significant difference was found between:

1. High-AQ males and Low-AQ males to painful pictures in Visual Task (p = 0.491);
2. High-AQ males and Low-AQ males to non-painful stimuli in both Auditory Task (p = 0.215) and Visual Task (p = 0.278);
3. High-AQ females and Low-AQ females to painful stimuli in both Auditory Task (p = 0.522) and Visual Task (p = 0.925);
4. High-AQ females and Low-AQ females to non-painful stimuli in both Auditory Task (p = 0.903) and Visual Task (p = 0.218).

### Pain intensity rating

For the main effect of Stimulus Type [F(1,116) = 2049.559, p < 0.001, η^2^ = 0.946], average pain intensity ratings was higher for painful stimuli (6.09±0.08) than non-painful stimuli (1.72±0.05). For the main effect of Modality [F(1,116) = 11.717, p = 0.001, η^2^ = 0.092], average pain intensity ratings was higher for voices (4.01±0.05) than pictures (3.81±0.54). For the main effect of Gender [F(1,116) = 5.831, p = 0.017, η^2^ = 0.048], average pain intensity ratings was higher for males (4.02±0.06) than for females (3.80±0.06). The interaction of Autistic Traits × Gender [F(1,116) = 4.039, p = 0.047, η^2^ = 0.034] indicated that the average pain intensity ratings of Low-AQ males (4.12±0.09) was higher than that of Low-AQ females (3.72±0.09) (p = 0.002), whereas no significant difference was found between High-AQ males (3.92±0.09) and High-AQ females (3.88±0.09) (p = 0.773). No other main effect or interaction effect was significant (all p-values>0.05).

### Emotional reaction

For emotion reaction, the main effect for Stimulus Type [F(1,116) = 313.848, p < 0.001, η^2^ = 0.730] indicated painful stimuli (6.53±0.08) were judged to be more unpleasant than non-painful stimuli (4.49±0.07). The main effect of Gender [F(1,116) = 4.976, p = 0.028, η^2^ = 0.041] indicated the average emotional reactions were more negative for males (5.62±0.07) than females (5.40±0.07). No other main effect or interaction effect was significant (all p-values>0.05).

## Discussion

The main goal of this study was to explore the potential relationship between responses to others’ pain and the autistic phenotype in TD adults across stimulus domains. In order to test this goal, we used two tasks: the Visual Task assessing the responses to others’ pain from pictures and the Auditory Task assessing the responses to others’ pain from voices. In summary, we found that the High-AQ group was less accurate than the Low-AQ group in identifying others’ pain, and High-AQ males judged others’ pain much slower than Low-AQ males, especially in auditory modality. These findings demonstrated that, compared to the Low-AQ individuals, individuals with High-AQ generally experienced difficulties in recognizing others’ pain correctly; and compared to controls, High-AQ males required more time to do so, especially in response to others’ painful voices.

### Reduced accuracy in the perception of others’ pain in High-AQ group

The current finding documented a reduced ACC in response to others’ pain in the High-AQ group, which indicated the ability to recognize others’ pain is related to the extent of autistic traits. This result was in line with the previous studies on emotional processing in individuals with autistic traits [20] and ASD [21, 22] showing an impaired recognition of some negative emotions (e.g., sadness, disgust and anger).

Clearly, individuals with autistic traits share similar traits with individuals with ASD and they usually engage less in social interactions than control groups [10]. Maybe the reduced experience in social interactions, specifically in noticing and identifying others’ pain, was a source of the observed altered responses in this group.

### High-AQ males’ responses to others’ pain

When analyzing males and females jointly, we found that the High-AQ and Low-AQ groups showed comparable RTs, pain intensity ratings, and emotional reactions in both visual and auditory tasks. However, analyzing the performance of males and females separately revealed interesting differences. Compared to Low-AQ males, High-AQ males showed slower identification of others’ pain, whereas females did not exhibit any difference between groups. Note that, as detailed in the “Participants” section, the present interaction cannot be attributed to differences between males and females in terms of AQ scores or ages.

This finding was in line with research suggesting that identification of others’ feelings and thought would exhibit more difficulties in males with autistic traits than in females with equivalent traits [23, 24]. Furthermore, our results were qualitatively similar to deficits observed in clinical groups. For example, one functional neuroimaging (fMRI) study did not find a fundamental impairment in total ASD participants but stronger difficulties in responses to others’ feelings for ASD males [25].

### High-AQ males’ responses to others’ pain in different modality

Past visual research has shown that, when participants with ASD observed others’ pain, they exhibited comparable subjective pain intensity ratings [8], unpleasantness ratings [8, 9], mu rhythms suppression [9], and hemodynamic responses in brain areas associated with pain sharing [26] with control groups. Consistent with these findings, this study reported similar results in the visual modality: High-AQ and Low-AQ groups displayed comparable RTs, pain intensity ratings, and unpleasantness ratings to painful pictures.

In the auditory modality, participants in the High-AQ group exhibited similar pain intensity ratings and unpleasantness ratings as participants in the Low-AQ group. This result was in line with previous research that found no evidence of a fundamental emotion recognition deficit in the ASD group on both the visual and auditory modality [27]. Interestingly, High-AQ males exhibited slower reactions than Low-AQ males, whereas females in the High-AQ and the Low-AQ groups did not show this pattern. This result supported the notion that High-AQ males may show lower fluidity in responses to others’ feelings [24]. The result was arguably due to High-AQ males orienting to the threat value of others’ pain in the voices, which delayed their responses. The “threat value of pain hypothesis” posits noticing others’ pain can be directly related to the perception of threat and danger and activates an early, threat-detection system [28]. Note that, the pain intensity of the auditory stimuli was rated significantly higher than visual stimuli. High-AQ males may be more vulnerable to distraction because of the threat aspect of others’ pain noticed in the voice.

Despite its possible implications, this study has two main limitations that may serve as foundations for next research. First, the participants in our study were university students. Accordingly, it is not clear that similar results would be observable in other populations. Indeed, confounding variables such as age and education level may play an important role in other populations, thus a future study might explore the relationship between responses to others’ pain and the autistic traits with a more heterogeneous population. Second, because reactions differed in response to others’ physical pain when presented with facial expressions [29], it will be necessary to explore whether findings from this study could extend to emotional communication of painful stimuli.

## Conclusion

In this study, we used painful pictures and voices to examine the responses to others’ pain in TD individuals with autistic traits. We have found a reduced accuracy in identification of others’ pain in the High-AQ group in relation to the Low-AQ group. Moreover, compared to Low-AQ males, High-AQ males had longer RTs to others’ painful voices. However, RTs of females were not influenced by autistic traits and modality. Our findings suggest that a selective difficulty in identification of others’ pain in healthy adults is related to the extent of autistic traits, gender, and modality.

## Supporting information

S1 Appendix Auditory stimuli for auditory task. (DOC)

S2 Appendix Auditory stimuli for the training session. (DOC)

S1 File Data for every participant. (SAV)

S1 Table Descriptive statistics [Mean (SD)]. (DOC)
