# Supplementary material for: Responses to others’ pain in adults with autistic traits: The influence of gender and stimuli modality
Source: PLoS One. 2017 Mar 20;12(3):e0174109. doi: 10.1371/journal.pone.0174109 (PMC5358845; doi:10.1371/journal.pone.0174109)
Supplement: S1 Appendix — (DOC) [file pone.0174109.s001.doc]

**Appendix 1. Auditory Stimuli for Auditory Task**

|  | **Painful voices** | | | **Non-painful voices** | | |  |
| --- | --- | --- | --- | --- | --- | --- | --- |
|  | **Content** | **Waveform** | **Duration**  **(msec)** | **Content** | **Waveform** | **Duration**  **(msec)** |  |
| **Male Speaker** | 烫伤  (Scalding) | 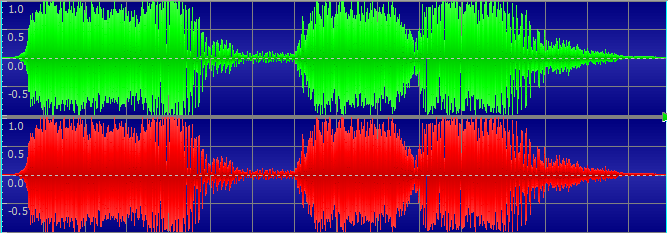 | 795 | 瘙痒  (itching) | 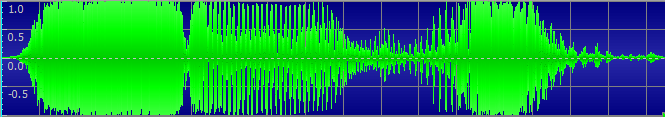 | 876 |  |
|  | 打伤  (pounding) | 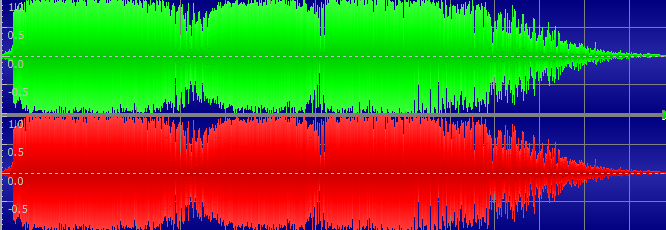 | 741 | 潮湿  (moist) | 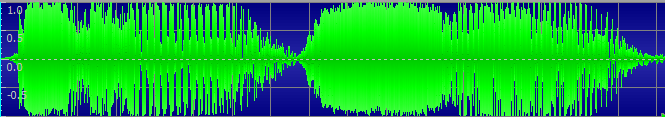 | 861 |  |
|  | 夹伤  (crunching) | 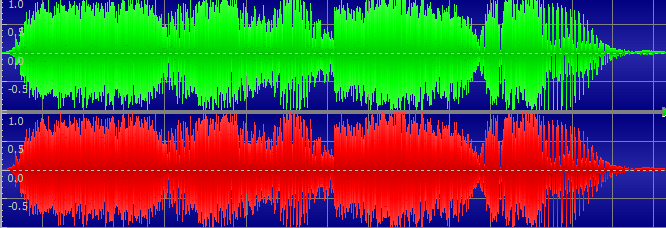 | 816 | 酷热  (hot) | 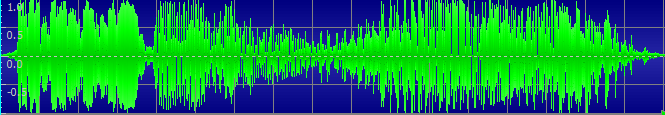 | 853 |  |
|  | 烧伤  (burning) | 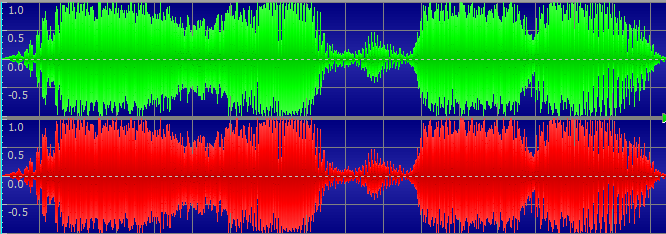 | 817 | 凉爽  (cool) | 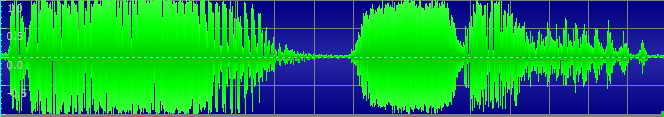 | 848 |  |
|  | 灼伤  (burnable) | 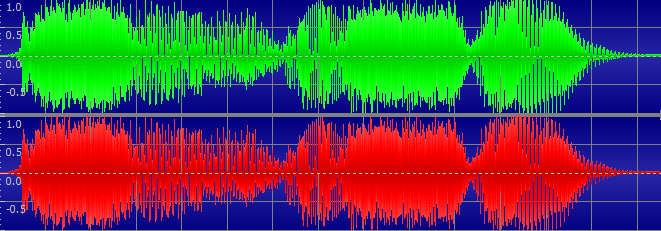 | 730 | 舒服  (comfort) | 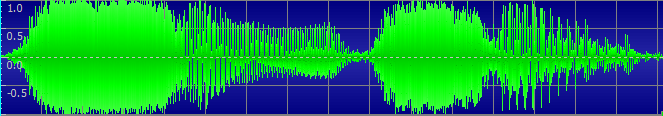 | 810 |  |
|  | 扎伤  (throbbing) | 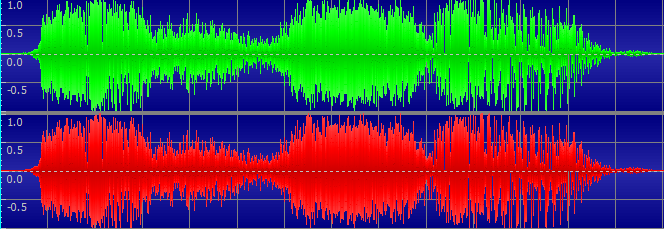 | 703 | 挠痒  (itching) | 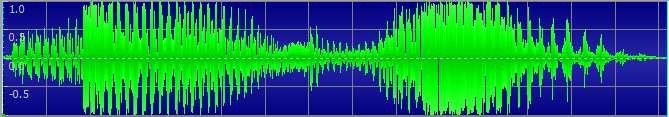 | 760 |  |
|  | 刺骨  (stabbing) | 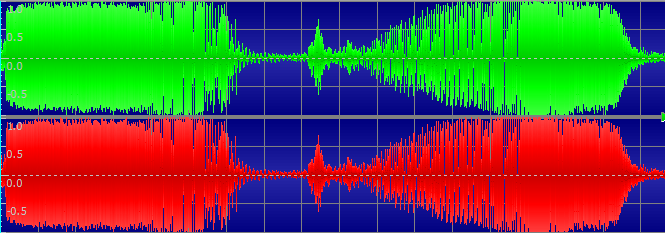 | 883 | 冰冷  (cold) | 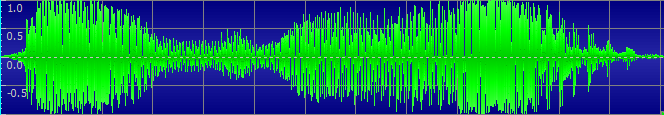 | 655 |  |
|  | 刺痛  (gnawing) | 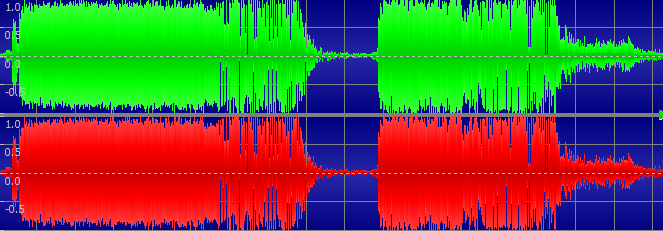 | 864 | 打湿  (damp) | 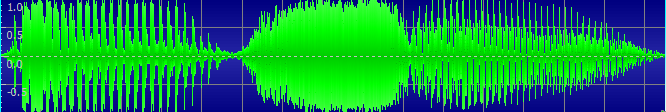 | 826 |  |
|  | 剧痛  (sore) | 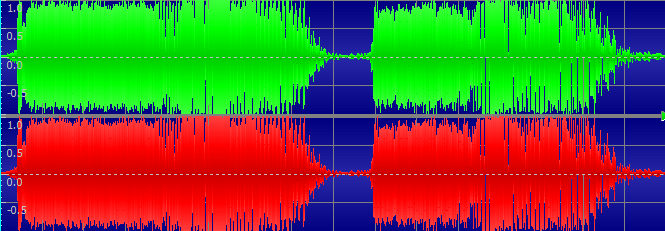 | 799 | 温暖  (warm) | 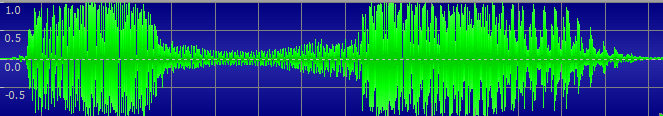 | 767 |  |
|  | 阵痛  (twitching) | 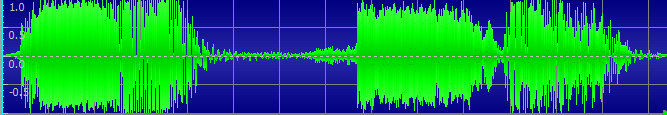 | 721 | 干扰  (distraction) | 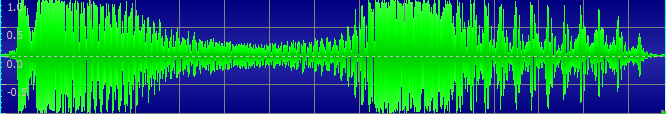 | 741 |  |
|  | 绞痛  (angina) | 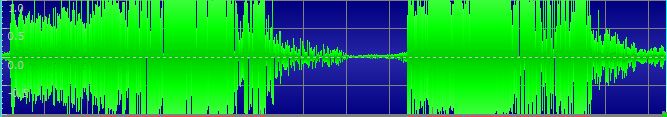 | 771 | 淋湿  (wet) | 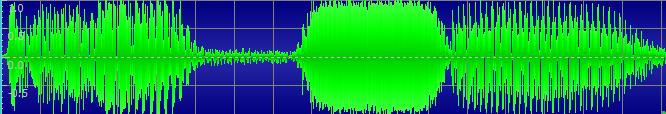 | 857 |  |
|  | 伤痛  (hurting) | 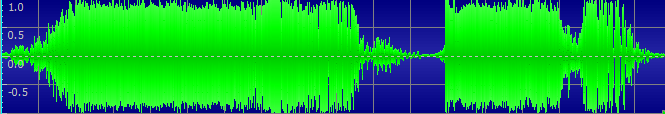 | 894 | 热晕  (sweltering) | 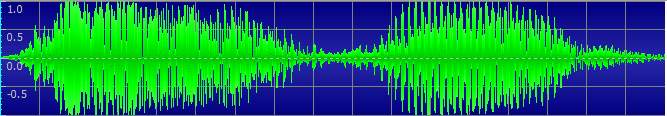 | 851 |  |
|  | 割伤  (cutting) | 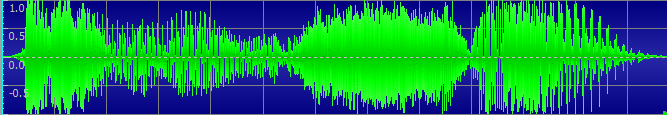 | 638 | 冻着  (cold) | 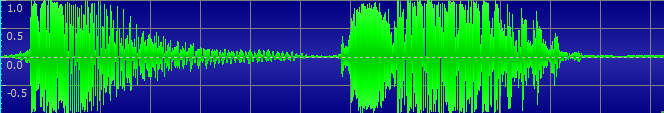 | 665 |  |
|  | 疼痛  (aching) | 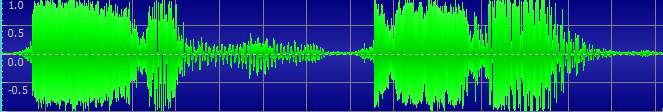 | 763 | 日晒  (basking) | 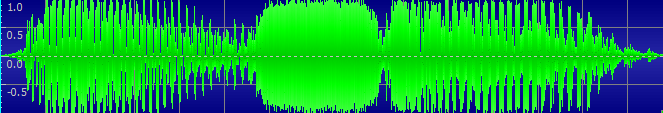 | 742 |  |
|  | 痛处(traumatic) | 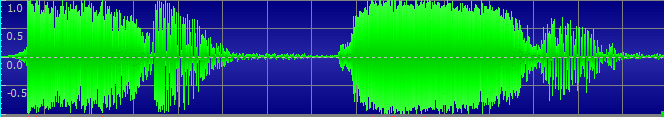 | 748 | 风吹  (blowing) | 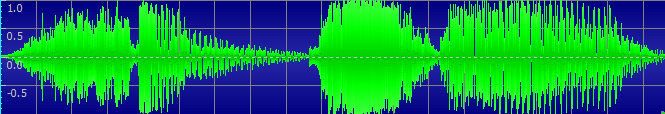 | 931 |  |
| **Female Speaker** | 烫伤  (Scalding) | 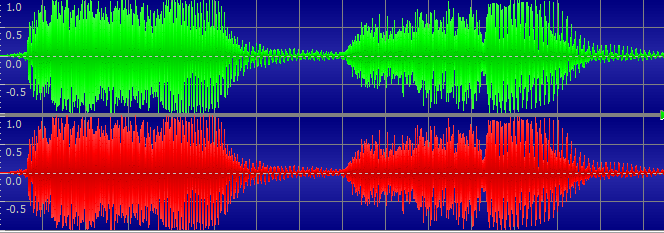 | 775 | 瘙痒  (itching) | 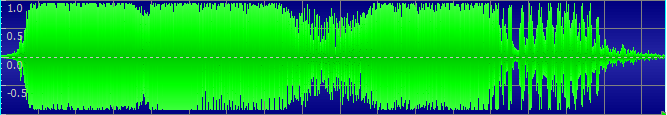 | 881 |  |
|  | 打伤  (pounding) | 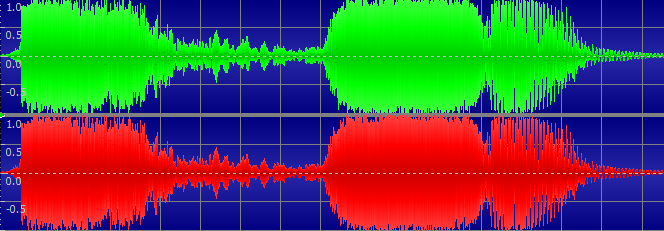 | 827 | 潮湿  (moist) | 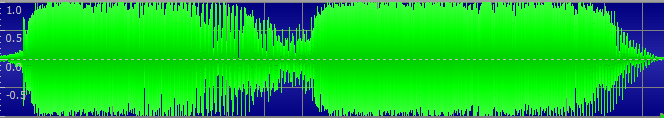 | 879 |  |
|  | 夹伤  (crunching) | 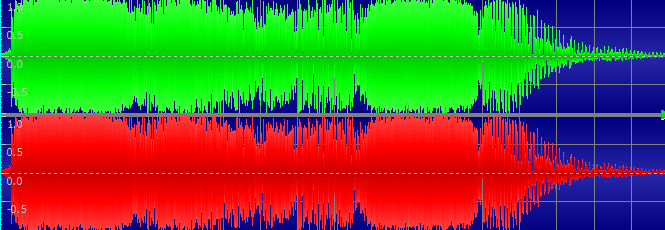 | 896 | 酷热  (hot) | 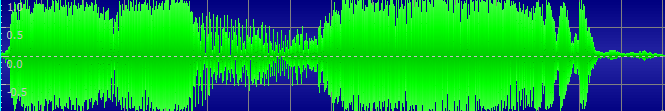 | 804 |  |
|  | 烧伤  (burning) | 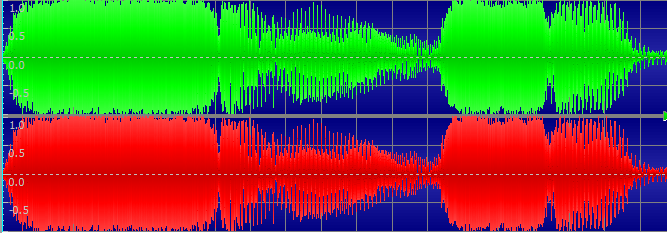 | 938 | 凉爽  (cool) | 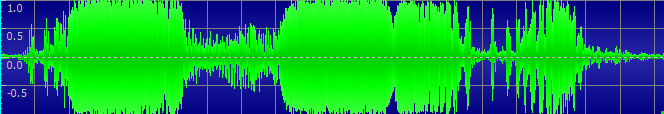 | 966 |  |
|  | 灼伤  (burnable) | 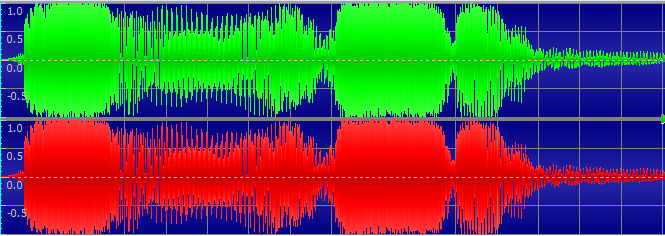 | 803 | 舒服  (comfort) | 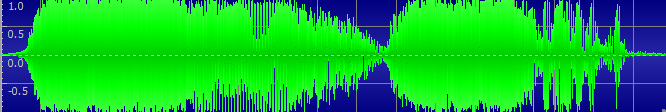 | 842 |  |
|  | 扎伤  (throbbing) | 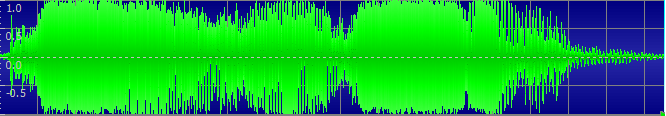 | 877 | 挠痒  (itching) | 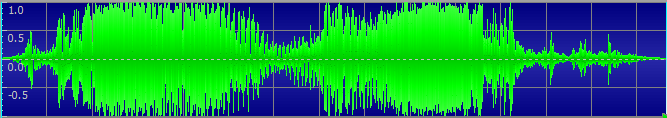 | 733 |  |
|  | 刺骨  (stabbing) | 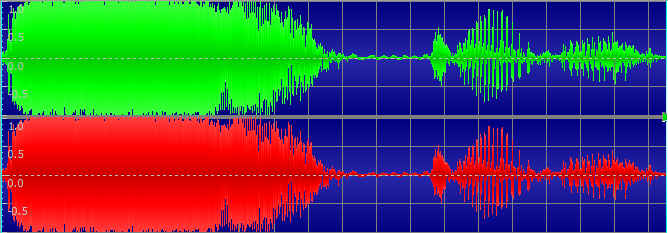 | 976 | 冰冷  (frozen) | 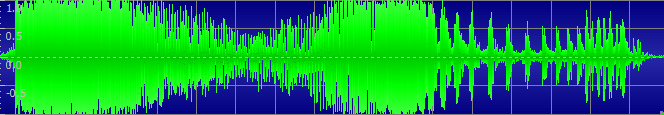 | 844 |  |
|  | 刺痛  (gnawing) | 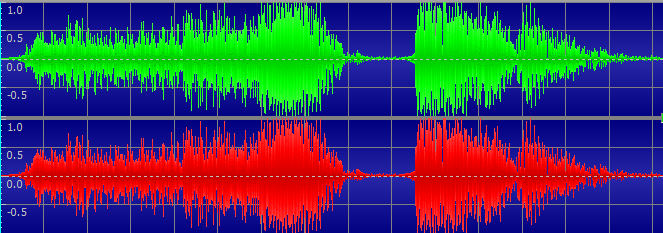 | 765 | 打湿  (damp) | 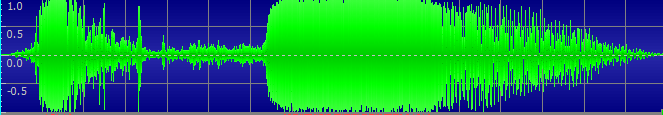 | 797 |  |
|  | 剧痛  (sore) | 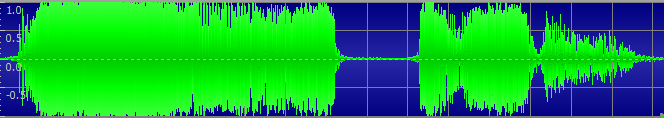 | 814 | 温暖  (warm) | 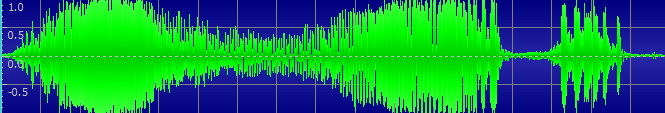 | 846 |  |
|  | 阵痛  (twitching) | 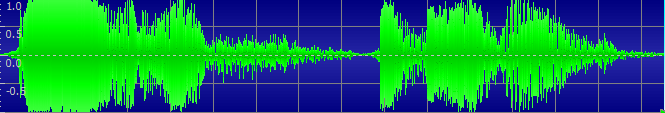 | 777 | 干扰  (distraction) | 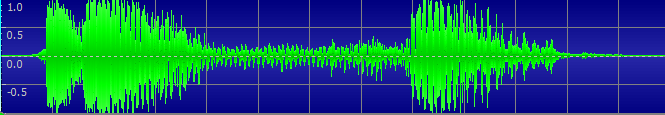 | 645 |  |
|  | 绞痛  (angina) | 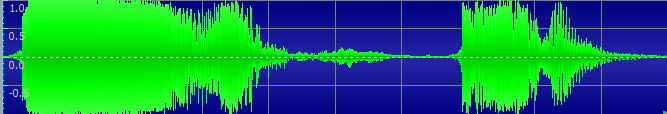 | 1000 | 淋湿  (wet) | 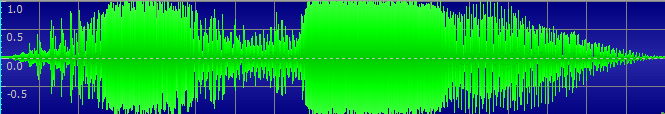 | 850 |  |
|  | 伤痛  (hurting) | 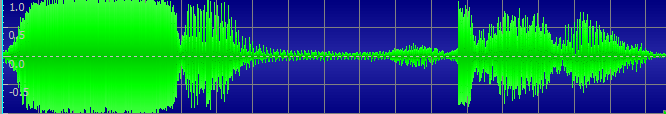 | 930 | 热晕  (hot) | 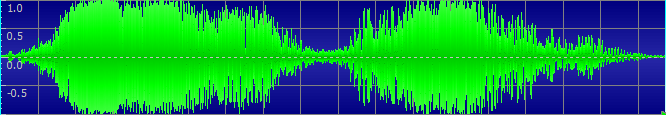 | 887 |  |
|  | 割伤  (cutting) | 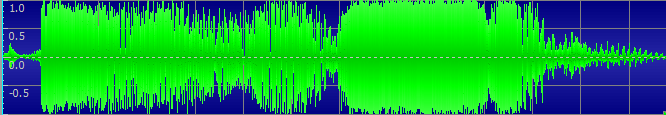 | 690 | 冻着  (cold) | 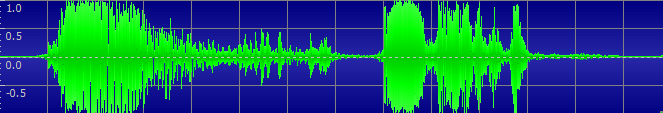 | 693 |  |
|  | 疼痛  (aching) | 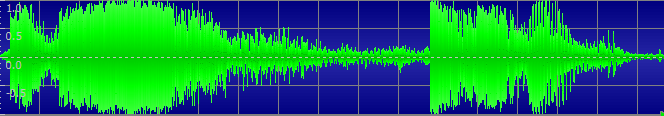 | 834 | 日晒  (basking) | 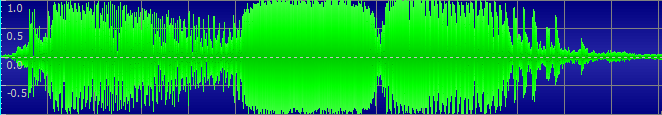 | 707 |  |
|  | 痛处(traumatic) | 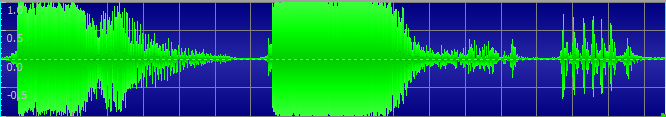 | 930 | 风吹  (blowing) | 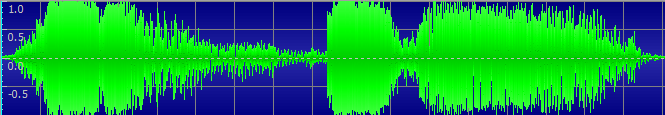 | 855 |  |

Note. Appendix 1 provides voices used in the study with corresponding English translations (in brackets), waveforms, and durations
