# Supplementary material for: Responses to others’ pain in adults with autistic traits: The influence of gender and stimuli modality
Source: PLoS One. 2017 Mar 20;12(3):e0174109. doi: 10.1371/journal.pone.0174109 (PMC5358845; doi:10.1371/journal.pone.0174109)
Supplement: S2 Appendix — (DOC) [file pone.0174109.s002.doc]

**Appendix 2. Auditory Stimuli for the Training Session**

|  | **Painful voices** | | | **Non-painful voices** | | |  |
| --- | --- | --- | --- | --- | --- | --- | --- |
|  | **Content** | **Waveform** | **Duration**  **(msec)** | **Content** | **Waveform** | **Duration**  **(msec)** |  |
| **Male Speaker** | 抓伤(scratching) | 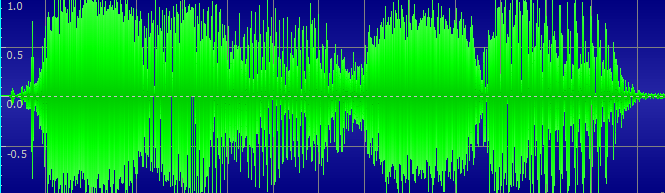 | 731 | 苦涩  ( bitter) | 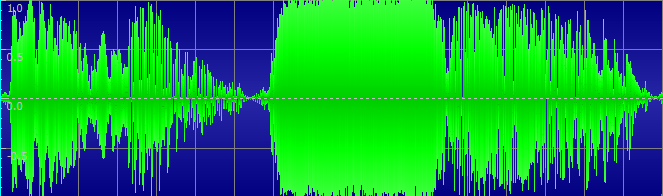 | 854 |  |
|  | 胀痛(swelling) | 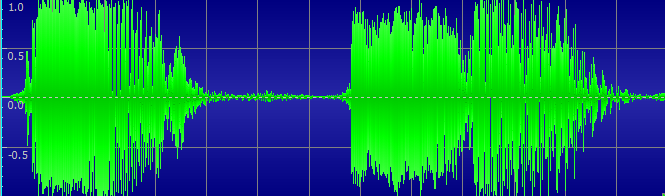 | 649 | 闷热  (stuffy) | 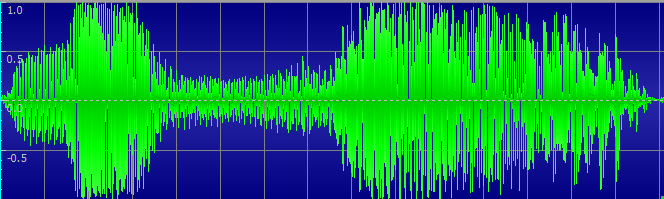 | 757 |  |
| **Female Speaker** | 抓伤(scratching) | 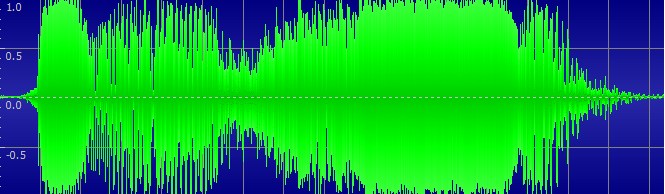 | 819 | 苦涩  ( bitter) | 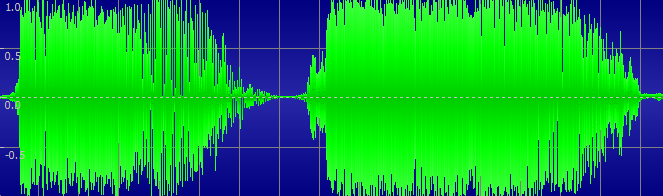 | 829 |  |
|  | 胀痛(swelling) | 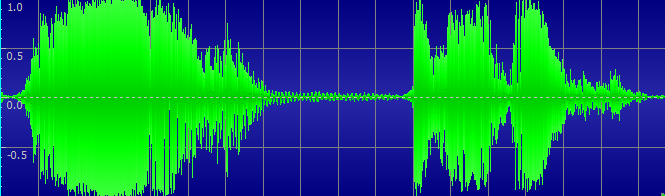 | 886 | 闷热  (stuffy) | 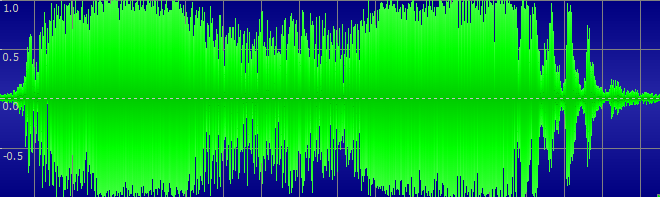 | 877 |  |

Note. Appendix 2 provides voices used in the training session with corresponding English translations (in brackets), waveforms, and durations
