# Supplementary material for: Responses to others’ pain in adults with autistic traits: The influence of gender and stimuli modality
Source: PLoS One. 2017 Mar 20;12(3):e0174109. doi: 10.1371/journal.pone.0174109 (PMC5358845; doi:10.1371/journal.pone.0174109)
Supplement: S1 Table — (DOC) [file pone.0174109.s004.doc]

| Autistic Traits | Gender | ACC | | | | RT (ms) | | | | Pain intensity ratings | | | | Emotional reactions | | | |
| --- | --- | --- | --- | --- | --- | --- | --- | --- | --- | --- | --- | --- | --- | --- | --- | --- | --- |
|  |  | Painful | | Non-painful | | Painful | | Non-painful | | Painful | | Non-painful | | Painful | | Non-painful | |
|  |  | Visual | Auditory | Visual | Auditory | Visual | Auditory | Visual | Auditory | Visual | Auditory | Visual | Auditory | Visual | Auditory | Visual | Auditory |
| High-  AQ | male | 0.921  (0.111) | 0.939  (0.094) | 0.949  (0.064) | 0.920  (0.101) | 898.66  (403.68) | 1119.82  (383.11) | 923.54  (426.12) | 1122.15  (372.74) | 6.05  (0.92) | 6.05 (1.34) | 1.77  (1.04) | 1.79  (0.50) | 6.45  (1.06) | 6.50  (1.27) | 4.60  (1.06) | 4.82  (0.91) |
|  | female | 0.949  (0.046) | 0.902  (0.130) | 0.958  (0.049) | 0.914  (0.109) | 891.50  (292.99) | 1058.56  (330.59) | 1081.57  (459.70) | 1117.32  (396.00) | 5.97  (1.02) | 6.24  (1.15) | 1.47  (0.64) | 1.84  (0.63) | 6.54  (0.75) | 6.44  (0.85) | 4.10  (1.18) | 4.55  (0.78) |
| Low-  AQ | male | 0.944  (0.039) | 0.955  (0.079) | 0.931  (0.085) | 0.908  (0.117) | 843.54  (225.36) | 903.38  (179.48) | 1048.41  (543.19) | 1007.28  (303.59) | 6.17  (0.91) | 6.53  (1.01) | 1.76  (0.97) | 1.99  (0.59) | 6.73  (0.84) | 6.92  (1.04) | 4.56  (1.09) | 4.41  (1.05) |
|  | female | 0.959  (0.044) | 0.951  (0.044) | 0.944  (0.094) | 0.871  (0.162) | 889.13  (301.23) | 1006.80  (310.19) | 937.17  (320.15) | 1105.84  (361.24) | 5.76  (0.65) | 5.97  (0.83) | 1.49  (0.47) | 1.65  (0.51) | 6.40  (0.86) | 6.27  (0.81) | 4.38  (1.12) | 4.53  (0.93) |

**Table. Descriptive statistics [Mean (SD)].**
